# Signe de « la dent molaire »: aspect caractéristique en IRM du syndrome de Joubert

**DOI:** 10.11604/pamj.2015.22.127.7971

**Published:** 2015-10-13

**Authors:** Manel Jellouli, Tahar Gargah

**Affiliations:** 1 Service de Pédiatrie, Hôpital Charles Nicolle, Tunis, Tunisie

**Keywords:** Syndrome de Joubert, néphronophtise, insuffisance rénale, Joubert syndrome, nephronophthisis, renal failure

## Image en medicine

Le syndrome de Joubert est une affection génétique rare, de transmission autosomique récessive, caractérisé par une malformation congénitale du tronc cérébral et une agénésie ou une hypoplasie du vermis cérébelleux entraînant des troubles respiratoires, un nystagmus, une hypotonie, une ataxie et un retard du développement moteur. L'imagerie par résonance magnétique permet de poser le diagnostic en mettant en évidence le “signe de la molaire” (Molartoothsign). Nous rapportons l'observation d'une fille âgée de 2 ans 4 mois, issue d'un mariage consanguin 2^éme^ degré, hospitalisée pour exploration d'un retard psychomoteur. A l'examen, elle présentait une hypotonie, la marche n’était pas encore acquise. Elle avait un syndrome cérébelleuxet des mouvements anormaux des yeux à type de nystagmus. L'image par résonnance magnétique cérébrale a révélé une hypoplasie du vermis cérébelleux, lespédoncules cérébelleux supérieurs étaient horizontalisés et épaissisavec aspect de «dent molaire» en faveur d'un syndrome de Joubert. L’évolution était marquée par l'installation d'un retard de croissance avec un syndrome polyuropolydipsique. A la biologie, une insuffisance rénale chronique progressive était identifiée. L’étude génétique n'a pas trouvé de mutation du gène NPHP1. Le diagnostic de néphronophtise était retenu devant l'association d'insuffisance rénale progressive avec le syndrome de Joubert.

**Figure 1:**
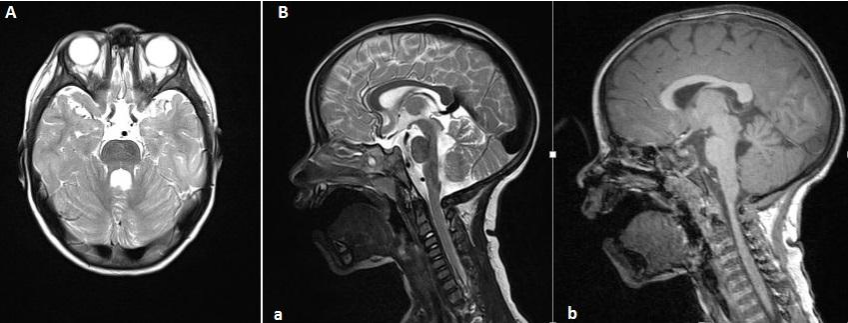
(A) IRM cérébrale en coupe sagittale montrant une hypoplasie vermienne prédominant à l’étage supérieure; (B) IRM cérébrale en coupe axiale en T1 et T2 montrant l’élargissement des pédoncules cérébelleux supérieurs avec l'aspect caractéristique en « dent de molaire »

